# Utilizing User Preferences in Designing the AGILE (Accelerating Access to Gender-Based Violence Information and Services Leveraging on Technology Enhanced) Chatbot

**DOI:** 10.3390/ijerph20217018

**Published:** 2023-11-03

**Authors:** Anne Ngũnjiri, Peter Memiah, Robert Kimathi, Fernando A. Wagner, Annrita Ikahu, Eunice Omanga, Emmanuel Kweyu, Carol Ngunu, Lilian Otiso

**Affiliations:** 1 LVCT Health Kenya, Nairobi P.O. Box 19835-00202, Kenya; anne.ngunjiri@lvcthealth.org (A.N.); robert.kimathi@lvcthealth.org (R.K.); annrita.ikahu@lvcthealth.org (A.I.); eunice.omanga@lvcthealth.org (E.O.); lilian.otiso@lvcthealth.org (L.O.); 2 Graduate School, University of Maryland, 620 W. Lexington Street, Baltimore, MD 21201, USA; 3 School of Social Work, University of Maryland, 525 W. Redwood Street, Baltimore, MD 21201, USA; fernando.wagner@ssw.umaryland.edu; 4 Faculty of Information Technology, Strathmore University, Nairobi P.O. Box 59857-00200, Kenya; ekweyu@strathmore.edu; 5 Department of Health, Nairobi City County, Nairobi P.O. Box 30075-00100, Kenya; ngunucarol@yahoo.com

**Keywords:** chatbot, violence, adolescent health

## Abstract

Introduction: Technology advancements have enhanced artificial intelligence, leading to a user shift towards virtual assistants, but a human-centered approach is needed to assess for acceptability and effectiveness. The AGILE chatbot is designed in Kenya with features to redefine the response towards gender-based violence (GBV) among vulnerable populations, including adolescents, young women and men, and sexual and gender minorities, to offer accurate and reliable information among users. Methods: We conducted an exploratory qualitative study through focus group discussions (FGDs) targeting 150 participants sampled from vulnerable categories; adolescent girls and boys, young women, young men, and sexual and gender minorities. The FGDs included multiple inquiries to assess knowledge and prior interaction with intelligent conversational assistants to inform the user-centric development of a decision-supportive chatbot and a pilot of the chatbot prototype. Each focus group comprised 9–10 members, and the discussions lasted about two hours to gain qualitative user insights and experiences. We used thematic analysis and drew on grounded theory to analyze the data. Results: The analysis resulted in 14 salient themes composed of sexual violence, physical violence, emotional violence, intimate partner violence, female genital mutilation, sexual reproductive health, mental health, help-seeking behaviors/where to seek support, who to talk to, and what information they would like, features of the chatbot, access of chatbot, abuse and HIV, family and community conflicts, and information for self-care. Conclusion: Adopting a human-centered approach in designing an effective chatbot with as many human features as possible is crucial in increasing utilization, addressing the gaps presented by marginalized/vulnerable populations, and reducing the current GBV epidemic by moving prevention and response services closer to people in need.

## 1. Introduction

Gender-based violence (GBV) is a global challenge and a human rights issue that continues to disproportionately affect vulnerable populations, including adolescents, young girls, and sexual and gender Minorities, owing to its deep entrenchment in traditions, cultures, and social institutions [1,2]. Currently, one out of three women undergo sexual or physical abuse by their partners or non-partner acquaintances at least once in their lifetime [3], and the same proportion of women’s homicides are reported cases of intimate partner violence [4]. At the same time, 75% of GBV survivors are likely to develop trauma [5,6,7]. Sexual and gender minorities communities also continue to experience victimization, increasing their chances of suicide [8,9]. Some societies and survivors justify the acts of perpetration, making GBV a complex discussion and demonstrating the need for strategies that reduce, if not eradicate, the vice and a multifaceted comprehension of GBV and its effects in society [10,11,12].

Adolescent girls ages 10–19 years and young women 20–24 years (AGYW) continue to be disproportionately affected by GBV compared to boys [13,14,15]. Adolescence represents the age at which GBV begins [16,17], with 35% of adolescent girls and young women globally having experienced GBV. Furthermore, the number is projected to rise in low- and middle-income settings [18,19]. GBV rates are equally high in Kenya, with statistics indicating that females are at higher risk (16%) of experiencing GBV before attaining the age of 18 years compared to males (6%), while up to 62% of girls will have experienced multiple counts of GBV by the time they reach 18 [20]. Overall, 39% of married women and 9% of men aged 15–49 report having experienced spousal physical or sexual violence [21].

Despite interventions, the GBV statistics still remain persistently high [18,21,22]. Global pandemics have been documented to increase GBV prevalence among adolescent girls and young women (AGYW) and other vulnerable groups [23,24]. Other factors include stigma and fear associated with GBV affecting reporting and response, low levels of education among AGYW, patriarchal societies that encourage female oppression, limited GBV knowledge and awareness age, and poverty [25,26,27], increasing the risk of this category to GBV. Sexual and gender minorities, including the transgender community, also face disproportionate GBV levels at the hands of intimate partners and strangers and are at an elevated risk of homicide, sexual assault, and sexual harassment due to their sexuality [8,9,28]. Beyond physical assault, sexual and gender minorities in Kenya are often discriminated against and suffer from high levels of stigma [28,29]. The group is deprived of social recognition, family support, employment opportunities, and education, with most of the GBV cases in the category going unreported [28,30]. The lack of access to justice, discriminatory laws, unequal protection by the state, and lack of access to sexual reproductive and mental health for this vulnerable population exacerbate these violations [31,32,33]. 

For the vulnerable groups, one common issue is the lack of access to timely, accurate information on what GBV constitutes, where to seek services, and what to expect at service delivery points if they experience GBV [20,21,28,30]. Those who experience GBV do not disclose (muted response); if they do, they receive suboptimal services or limited referrals for support and care that are not client-centered care due to institutional system barriers [20,34]. The knowledge gaps limit adolescents’ and young people’s ability to realize and claim their sexual and reproductive health rights and make informed health decisions [27]. Therefore, there is a need to identify and upscale innovative strategies that can potentially address GBV through awareness creation and providing timely and holistic services [34]. Evidence-based approaches, including the uptake of digital interventions, have been proven critical in improving healthcare coverage in Kenya [35]. Recent years have seen an upward trajectory in using digital services to enhance service accessibility and delivery [35,36,37,38,39,40]. Chatbots have historically been critical in expanding care coverage by making information more accessible to the target population [37,41,42,43]. Chatbots are messaging interfaces that work by imitating human interactions to the highest possible levels, allowing users to receive intelligent assistance without human intervention [39,40]. Anti-violence bots have been shown to address GBV issues [44], and COVID-19 bots to increase coverage of COVID-19 information during the pandemic’s peak [45]. Conversation agents have also been used to effectively provide mental health services [37,46]. Research is ongoing to understand user preferences and improve digital platform usability and functionality [42,43,44]. Incorporating such virtual assistants in GBV prevention and response is an innovative strategy that can benefit a significant percentage of the vulnerable population in Kenya who have access to digital devices and address healthcare limitations. Participatory research is crucial in designing interventions that work for the population of study through and inquiry collaboration with the target beneficiaries to identify their issues and develop functional structure that address those needs [47,48,49,50,51].

To this end, LVCT Health, working with the University of Maryland and the Kenya Ministry of Health, embarked on the development of the AGILE chatbot for adolescents, women, and people from the LGBTQ community who wish to access and receive GBV services. The AI chatbot will provide GBV-related information and advocate for health-seeking behavior change amongst the users. In addition, the AGILE chatbot intends to deliver tailored self-care conversations to GBV survivors and those at risk. The chatbot will be hosted on the one2one™ digital platform that provides information on sexual and reproductive health, mental health, GBV information, tele-counseling, and referral to services that meet the needs of the survivors. This exploratory qualitative study aimed to understand potential users’ preferences, expectations, acceptability, and motivation for using such a bot. These findings will help the AGILE chatbot development by applying a human-centered approach and its implementation. 

## 2. Methods

### 2.1. Design and Recruitment of Participants

We used an exploratory qualitative approach in the AGILE chatbot knowledge generation on GBV-related content We conducted a series of exercises to gather valuable insights and feedback. 

The study adopted the same panel of participants during the four steps of prototype development. The participants consisted of (1) adolescent boys and girls, emancipated minors aged 17 years, and those aged 18 and 19 years; (2) young women aged 20–24 years; (3) young men aged 20–24 years; (4) those who identify as sexual minorities (LGBTQ). The youth department staff at LVCT Health reached out to potential participants aged 17–19 years using a snowballing technique to recruit them into the study. The youth were recruited from urban and peri-urban areas as internet-enabled devices are ubiquitous in these areas. These youth tend to be more tech-savvy and would likely benefit from a chatbot intervention.

Participants were provided with an information sheet on the study and the purpose of the data collection exercise. The inclusion criteria were for the participant to be either an emancipated minor, age 17, or age 18 and above, capable of consenting to the study. The eligible participants signed a consent form before engagement in which they were explicitly informed that their participation would involve audio recording, note taking and that their data would be used in production of publications. Additionally, they were notified of their right to stop their engagement at any point during the data collection process, without any consequences. The focus group discussions (FGDs) consisted of 10 participants each. The participants were provided with reimbursement for their transportation expenses as well as lunch.

### 2.2. Data Collection

We commenced with a comprehensive desk review of the LVCT Health one2one™ digital platform. The review aimed to retrieve frequently asked questions by GBV survivors or those at risk of GBV as they seek support from the tele-counselors. We retrieved messages from the call center, SMS, and WhatsApp logs, covering two years—2020 to 2022. This objective was to compile likely questions and information to be probed during discussions with the target population. 

Subsequently, and prior to the prototype development, we initiated the first round of focus group discussions (FGDs). The objective of this exercise was to gather participant insights on chatbot design and functionality for addressing GBV and sexual reproductive health, including the participants’ understanding of chatbots; and their opinion on the type of information and services on GBV and sexual and reproductive health to be provided by the chatbot; and their preference on how they would like the chatbot to communicate with them (language, features, emojis). We developed topic guides for the FGDs consisting of open-ended questions that explored these concepts During the FGDs, the World Café methodology [52] was incorporated, a simple, practical format for hosting group dialogues. Four stations were set up, with two unique questions retrieved from the desk review. The questions were in English and translated into Kiswahili, and each group (of five participants) was asked to list down likely responses they wanted the chatbot to provide for the questions. 

After the FGDs, we conducted the prototype simulation exercise where the developers guided the participants on how to enter the chatroom and provided a demonstration. The objective of the prototype simulation exercise was to assess participant engagement, familiarity, and user experience with the AGILE chatbot, gathering feedback on its usability and effectiveness in responding to GBV-related questions. The participants were requested to converse with the AGILE chatbot by posting any GBV-related questions and gaining responses from the chatbot prototype. The simulation exercise lasted 20 min. This allowed the participants to interact with the AI bot. After the simulation, the participants completed an ‘Engagement Questionnaire for AGILE chatbot’, providing scores on their experience of the AGILE chatbot use. The engagement questionnaire tested the user experience for terms such as easy to use, engaging, informative, motivating, messy, complex, and innovative.

Finally, the data collection concluded with a round of FGDs that focused on the ‘prototype simulation’ participants’ experience. The discussions provided insights into the participants’ understanding of the AGILE chatbot prototype, the context they would use or access the chatbot for support, and any changes they would like to make to the chatbot’s functionality.

The FGDs were conducted in Nairobi, Kenya, a preferred choice which allowed the research team to engage with a diverse group of participants representing various backgrounds and perspectives within the urban setting. All FGDs conducted were audio recorded. Trained research assistants moderated the FGDs and simulation exercises and were involved in the transcription, data analysis, and validation. 

### 2.3. Data Validation and Analysis

The analysis aimed to uncover emerging themes and gain insights into the research questions. First, the audio files were transcribed, converting the spoken language into written text, which facilitated further analysis. Subsequently, data validation was conducted after the production of the transcripts from the audio files. Three research team members undertook a cross-verification exercise where the scripts were reviewed against the matching original audio file, and where necessary the transcripts were corrected. This was to ensure fidelity of the written content in the scripts, eliminating any discrepancies. 

The data validation process was followed by a systematic approach involving coding, categorization, and thematic analysis [53]. This rigorous process allowed for the identification of key themes and the extraction of meaningful insights from the qualitative data. The transcribed data were then subjected to coding, where meaningful segments were identified and assigned labels or codes. The coding process enabled the organization and structuring of the data for subsequent analysis. Three research team members with expertise in qualitative research were engaged in the coding framework development and had a shared understanding of the coding guidelines. The team conducted iterative coding sessions where each team member independently coded a subset of data, line by line. Meetings were held with the team to review and discuss the coding outcomes and resolve any discrepancies through consensus, as the coding framework was refined. Next, we conducted axial coding to identify connections between the coded segments and categorized into broader themes based on their content and relevance. The categorization process involved grouping similar codes together to identify patterns and commonalities within the data. This step helped identify the main themes that emerged from the transcribed discussions. Following the categorization, thematic analysis was conducted on the categorized codes. Thematic analysis is a qualitative research method used to identify patterns, analyze meanings, and report themes within a dataset [54]. The themes represent recurring topics or ideas that reflect the content and meaning of the FGDs. In this study, the researchers employed deductive content analysis, which involves applying a pre-determined coding framework or analysis matrix to the data [55]. This framework was informed by prior theoretical knowledge or existing models relevant to the research topic. By utilizing deductive content analysis, the researchers aimed to test concepts, categories, or theories within the context of the transcribed discussions. The results of the analysis provide a comprehensive understanding of the FGDs, aligning with the research objectives and contributing to the overall findings of the study.

### 2.4. Approval

The study was approved by AMREF Ethics and Scientific Review Committee (ref P1204/2022) and the National Commission for Science, Technology, and Innovation (ref P-22-17893) in Kenya.

## 3. Results

The analysis resulted in 14 salient themes composed of sexual violence and female genital mutilation, physical violence, emotional violence, intimate partner violence, sexual and reproductive health, mental health, help-seeking behaviors/where to seek support, who to talk to, and what information they would like, features of the chatbot, access to the chatbot, abuse and HIV, family and community conflicts, and information on LGBTQ issues. 

### 3.1. Functionality

#### 3.1.1. Defining Chatbots

The respondents’ knowledge of the chatbot was assessed by asking them to define a chatbot. Some respondents could define a chatbot, others had no information on chatbots, while others could only describe how a chatbot works.

*“I think a chatbot is a programmed robot. Let me put it like that. It is there to assist in case nobody is around to assist purely. So, like, for example, you wanted to call and talk to someone.”* *—FGDYW001-R4*

*“A chatbot is like a phone, but it has automated answers in which you ask questions concerning yourself or confidential questions which you can’t share with people out there, and then you get automated answers.”—FGDAGB003-R6*

*“A chatbot is a programmable AI (artificial intelligence) used to respond to people.”—FGDYW003-R2*

#### 3.1.2. Adolescent Experiences with Chatbots

On previous experiences with chatbots, the participants mentioned that they had interacted with other chatbots while purchasing goods online and on telecommunication platforms. Other adolescents also indicated they might have unknowingly interacted with the virtual assistants. Participants expressed both satisfaction and frustration.

*“TIMS for NTSA. I used it to reapply for a new driver’s license.”* *—FGDYW003-R2*

*“I have used one from KUCCPS (Kenya Universities and Colleges Central Placement Service). I was inquiring about placement, so I registered, but it was delayed a bit, so I had to chat, and the chat was immediate; it wasn’t personal, meaning it was some chatbot.”—FGDAB003-R9*

*“* *I think the experience is good to some extent because you can get firsthand help before you go to… for example, the FLO chat before you visit a gynecologist, which we know is expensive. You can get a… the first hand like… it is a step. Like a step.”—FGDYW002-R1*

*“Annoying, due to the simple fact that occasionally some of the questions you may ask may be difficult to interpret, especially when you find maybe people have accents.”—FGDLGBTQ001-R1*

#### 3.1.3. Willingness to Use Chatbot

Respondents indicated the use of the chatbot depended on the need and the specific issues, suggesting perhaps a measure of the severity of their GBV experience that would lead them to seek support via a chatbot or not.

*“It depends; it depends not at every time. But it depends.”—FGDYW001-R1*

*“Yes, I can use it because not everyone knows everything. It is there to help; it can help me with what I don’t know.”—FGDAGB002-R2*

However, they indicated they would consider using the AGILE chatbot depending on the features and functionality of the chatbot.

*“It depends because of the efficiency… about the efficiency of the chatbot. Like, will I get the help I’m looking for? Will it be available? Will the response be what I’m looking for, or is this a robot that doesn’t understand? All these, yeah, the efficiency; it depends on the efficiency.”—FGDLGBTQ002-R3*

#### 3.1.4. Reduced Chances of Conflict or Disagreements Compared to Human Interactions

The respondents indicated various instances of conflict when seeking health care or help from adults, including parents and service providers, especially on matters related to GBV. Therefore, they would opt for the AGILE platform if it could understand GBV issues with minimal effects on user self-esteem.

*“Yes, I would use it; because of conflict, so many people don’t get along; I would like us to get along well.”—FGDAB003-R4*

*“Yes, I would use it because of the fear of talking to parents, you just use it, and they answer you directly.”—FGDAB003-R3*

### 3.2. Personalisation

#### 3.2.1. Confidentiality and Privacy

The respondents mentioned that they would prefer using chatbots due to the privacy and confidentiality of the platform compared to human interactions.

*“I feel the AGILE platform is a good chatbot because you can be afraid to speak out, but it gives you an opportunity as you are alone, and it’s someone you can’t see; you get automated answers. So, you say everything you feel, every question you have been asking yourself, finding answers, and you won’t feel ashamed because no one will judge you outside. It will be confidential, so I feel it is good.”—FGDAB003-R6*

*“…I would use the chatbot because, when you look at the world nowadays, it is easier to talk to someone else you don’t know or doesn’t recognize you because of confidentiality, honesty, and trust. I might be talking to someone, and they chase some clout with it the minute we fall out. So, confidentiality is one of the key things that would make someone use a chatbot.”—FGDLGBTQ002-R4*

#### 3.2.2. Accuracy of the Information Provided

The respondents also mentioned frequent challenges in getting accurate information on most GBV subjects forcing them to seek information from unreliable sources, including friends and online platforms.

*“Besides whatever they have said, I can use it… because of accuracy. I could have information about something, but that is not the exact information. I trust the chatbot that whatever it shows, there is something that has been researched, and it’s something that I can rely on. Also, confidentiality; some people don’t share anything with anyone. If something happens to them, they’d rather die with it, but they will never speak out. If there is this chatbot that I can type anything and it can give me almost a solution to my problem, I will relax.”—FGDAGB002-R4*

#### 3.2.3. Capacity to Address Existing Barriers to Healthcare 

The respondents were willing to embrace the chatbot if it could resolve healthcare barriers, including fear and stigma associated with seeking GBV services.

*“I feel the AGILE platform is a good chatbot because you can be afraid to speak out, but it gives you an opportunity as you are alone, and it’s someone you can’t see; you get automated answers. So, you say everything that you feel, every question you have been asking yourself, find answers, and you won’t feel ashamed because no one will judge you outside. It will be confidential, so I feel it is a good thing.”—FGDAB003-R6.*

*“First of all, this chatbot will help prevent mental harassment to prevent trauma where you can’t speak to someone because of what happened to you. It will also prevent you from feeling lonely; at least it will assist you in that if you had thought of committing suicide, it will have made you feel comfortable.”—FGDAB003-R8.*

### 3.3. Desired Content

#### 3.3.1. Sexual and Reproductive Health

On sexual and reproductive health, the respondents required information on contraception, sexually transmitted infections including HIV, information on pregnancy, menstruation, fertility, sexual health, sex toys, and information on safe abortion.

*“Which is the best contraceptive to use? I had sex with a person of unknown status. What do I do? If someone gets a discharge, what would be the problem? I need to get pregnant, and I don’t know what to do?”—FGDYW001-R1*

*“Can I access information about fertility and where? Then maybe I am experiencing a certain kind of discharge; what could be the cause? How do I get help?”—FGDLGBTQ002-R4*

*“What types of contraceptives are available for same-sex partners? How do I use lube? How do I wear a female condom?”—FGDLGBTQ002-R4*

#### 3.3.2. Mental Health Information

The respondents were keen on gaining more information from the chatbot on mental health topics, including symptoms, suicide, trauma, depression, mental GBV, and online counseling options.

*“I think it needs first to understand my type of mental problem. Then, it needs to tell me what is mental to understand what is mental.”—FGDYW001-R1.*

*“I feel like you should give me a… it should refer me to somebody immediately.”—FGDYW001-R4*

*“So, my other point was: Is this chatbot just going to be on issues that are happening now, or can it be something that happened ten years ago, maybe when I was a child, and I now need help because maybe this thing is affecting me now and I didn’t know, but now that I am older, I can maybe trace that it is because of this thing that happened when I was younger?”—FGDLGBTQ002-R8*

*“What can someone do if they are feeling suicidal? What is mental health? What is mental GBV?”—FGDLGTQ002-R3*

#### 3.3.3. Help-Seeking Behaviors

On help-seeking behavior)/where to seek support, the respondents wanted information on referrals, precautions & procedures, child support services, safe spaces, and queer-friendly and safe spaces.

*“In case of rape, what procedures to follow to ensure I’m physically and mentally safe?”—FGDAGB002-R5*

*“Which hospital near me is queer-friendly? What safe ways can I and my partner indulge in matters? Any safe spaces around me? Are there support groups for GBV?…* *They should keep you safe; you have reported him, and he has been arrested. Maybe they take you from where you were living to somewhere else.”—FGDLGBTQ002-R4*

#### 3.3.4. Community and Family Conflict

The respondents needed information on conflict resolution, general information, and family conflicts and conflicts within the community.

*“How can you finish conflicts in society? My family and I aren’t on good terms; what should I do?”—FGDAGB003-R4*

*How can I live with the people in the community? Will people love me again? FGDYW001-R10*

On information on sexual and gender minorities, the respondents expected the chatbot to address general questions on sexual and gender minorities, dating, safe places, and referrals.

*Is the place you referred me safe for my community (sexual and gender minorities)? Can I sue my partner, as our community’s union is considered illegal? —FGDLGBTQ002-R2*

*Why am I queer? Did something happen during my childhood that made me this way? Is something wrong with me?—FGDLGBTQ002-R9*

#### 3.3.5. Exclusions

The respondents also highlighted that the chatbot should not include personal identifiers, insensitive responses, questions on perpetrator details, or blaming responses and should be able to solve problems.

*Distributing blame, e.g., asking dressing code or time? FGDYW003-R3*

*“One of the things I wouldn’t want to get in a chatbot is judgment because why would there be, ‘What did you do to get this?”—FGDLGBTQ002-R4*

*You know most people don’t like to click these links. So I wouldn’t say I want to click the links. Yeah, it will be okay if you can tell me the complete information there. But redirecting me to another path, it’s a lot”—FGDLGBTQ001-R7.*

Participants articulated a need for accurate and comprehensive information on these subjects, highlighting the chatbot’s potential to bridge information gaps.

### 3.4. Usability and User Expectations

#### 3.4.1. User Expectations

The respondents also provided suggestions on the expected chatbot features, including the ability to understand multiple languages such as Swahili and sheng (adolescent cant-Swahili, English-based). Other offerings include responding in simple and easy-to-understand vocabulary and being available on various platforms, including web and app-based formats. In addition, the chatbot should possess emotional awareness, visual characteristics, confidentiality, a simple and descriptive name, integration with human help, round-the-clock services, and offline availability in case the users lack Wi-Fi or operate from a region without internet coverage and should be available in the lite version.

*“Something that will engage us as young people. And something that will give us the correct information. Yeah, not just information just because it is information. But correct information, and it should be easy to access it.”—FGDLGBTQ002-R1*

*“I feel it should communicate in both English and Swahili because some people are not perfect in English. They will understand Swahili more.”—FGDAGB002-R10*

*“I feel the information should be brief, short, and clear. There is no need for abbreviations, emojis…It should have sympathy words so long as they are appropriate and it tells you…Some people are in a situation whereby they want to be told something to feel good. It will relax them.”—FGDAGB002-R8*

*“According to me, I feel they have put in a little time. 8–10 is little, even if it’s every day. Something might happen at 11 p.m. or 6 a.m. when you want to help and don’t want to wait until tomorrow. But they have said you call from 8–10, so I feel the time is not very appropriate.”—FGDAGB002-R9.*

*“I would like for this chatbot to have confidentiality, first of all. For example, I have asked a question about something, and it should give me an answer which I will feel satisfied.”—FGDAGB003-R2*

#### 3.4.2. Respondents’ Expectations from the AGILE Chatbot

Regarding GBV, including female genital mutilation, the respondents expected the digital platform to be integrated with human interactions like a toll-free number. This is along with the interface to improve services, including precautions and procedures post SGBV, the referral pathways, reporting methods, risk assessment, safe houses reassurance and counseling services, and details of the act of perpetration.

*“In case of rape, what are the right procedures to ensure you are both physically and mentally safe?”—FGDAGB002-R5*

*[The chatbot should provide information about] What precautions and procedures should a GBV survivor undertake to ensure they continue to live a normal lifestyle? When you have been raped, what are the steps to follow? Regarding domestic violence, is it normal?”—FGDAGB002-R6*

In case of rape, what would you expect from the chatbot?

*[The following information] Do not shower, Do not wash the clothes, Give directions on what to do next, Refer the case, or connect me to the hotline. Call an emergency number.”—FGDAGB001-R6*

*“First, ask for the location—time of the incident. [then tell them] Do not shower. Go to the nearest health center and then go to the police station. Do you have anyone you can talk to? Go to the nearest police station so that they can refer you to the nearest health facility. It would be best if you kept the clothes for more investigation. The next question is if someone has been raped. It would help if you kept the clothes for more investigation. First, you should use polite language to approach the situation, e.g., sorry—the first precaution to take, e.g., not showering. Positive response, e.g., goes to the nearest police station and report. It would help if you visited the nearest health facility for more tests, e.g., HIV, pregnancy, etc. Seek guidance and counseling.”—FGDAGB001-R6*

**Figure 1:**
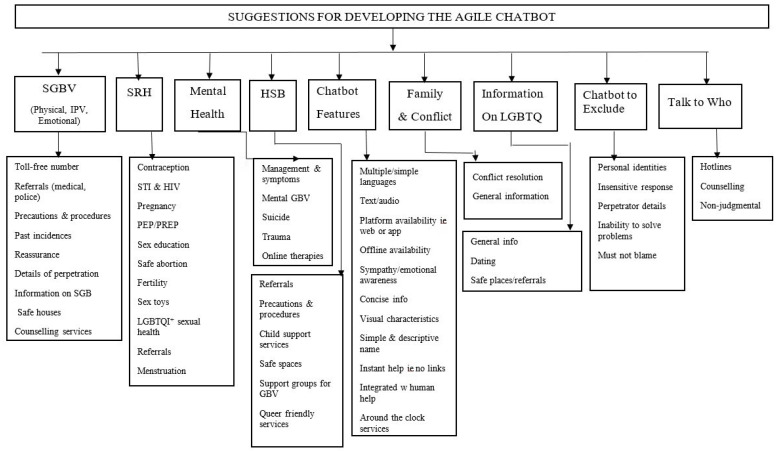
An illustration of the suggestions for developing the AGILE chatbot. SGBV—Sexual and Gender Based Violence; SRH—Sexual and Reproductive Health; HSB—Health Seeking Behaviour.

Based on the insights gathered from the FGDs, we developed a conceptual model that would provide a framework for the development of the AGILE chatbot content. The conceptual model, shown in Figure 1, captured the key topics that were identified by the participants during the FGDs. 

The model served as served as a roadmap to ensure that the chatbot’s development aligns with the expectations and needs expressed by the participants in the FGDs. This ensured that the AGILE chatbot would be user-centric and effectively address the concerns related to GBV, as identified through the FGD discussions. 

## 4. Discussion

Human-centered designs (HCDs) are crucial in developing innovative interventions by involving the beneficiaries in the development and implementation phases ensuring the users are at the core of the design process by understanding and incorporating their needs and preferences to establish products developed and embraced by the users [40,48,51,56]. HCDs must harness the values and priorities of users in designing interventions when creating digital platforms to enhance interaction and utilization. Although technology is vital in augmenting services, studies have identified little uptake and adherence to existing digital services attributed to user behavior, the design of the app, and the level to which human behavior is incorporated in the app [51,57,58,59,60]. Therefore, it is critical to obtain a comprehensive user insight, including the behavior and priorities of the targeted vulnerable population, before designing an app for their use. From the study findings, the willingness of the study participants to use the AGILE chatbot hinged on its efficiency and effectiveness, emphasizing the importance of functionality in shaping user adoption.

Studies have also demonstrated that the lack of HCD interventions hampers service delivery and utilization, leading to the underutilization of some critical services provided within general traditional health provision at community of facility setting. Studies identified that vulnerable people have a poor health-seeking attitude as they are hesitant to use traditional health services. Some reasons provided were at perceived complexity or discomfort, health provider level factors such as biases, stigma, negative attitudes and a lack of comprehensive information that bar vulnerable populations from reporting GBV cases and seeing support [61,62,63,64,65,66]. This underscores the significance of HCD interventions in healthcare which prioritize usability and accessibility. Discreet, personalized digital intervention for vulnerable populations can address these health-seeking barriers by eliminating the barriers and stigma related to seeking these services from a human provider. The AGILE chatbot aims to assist survivors or those at risk of violence by proactively helping them seek assistance and make informed decisions. It plays a crucial role in providing information and practical assistance to users, allowing them to access essential services and resources effectively. By acting as a facilitator, the AGILE chatbot ensures that users are provided with the necessary information and support to access the resources they require. This would significantly enhance access to information and assistance on sensitive topics—GBV, sexual and reproductive health and mental health—via a channel that is discreet, non-judgmental and easily accessible for users seeking information and support.

Another issue raised by the respondents of the present study on the features of a chatbot included privacy and confidentiality when seeking help on sensitive topics. Research on adolescents living with HIV also noted that this vulnerable population group is likely to forego health care associated with limited confidentiality leading to health detriments [67]. Other studies also demonstrated that lack of privacy hampers care-seeking and continuity among vulnerable populations [66]. A study investigating the credibility of chatbots indicates that users are more candid when confidential services are offered [58,59] and more straightforward on digital platforms compared to interactions with human service providers [60,61]. 

Other studies have illustrated that vulnerable populations prefer population-specific health provision approaches that are tailored to their specific needs and circumstances. [66,68,69]. In the context of digital health interventions, HCD is used to create solutions that address the unique challenges and preferences of different populations. Involving vulnerable populations in designing a chatbot that fits their needs is crucial in enhancing service provision and utilization [27,37,48,51]. This could potentially lead to a more extensive population reporting and being linked to GBV response services.

Studies have identified that vulnerable populations seek healthcare solutions from websites and peers, which can be misleading [67,70], increasing the chances of adopting sub-optimal interventions and associated risks [21,71]. Inadequately trained human service providers are also prone to errors. They can endanger the lives of users [72]. At the same time, effectively programmed chatbots are less likely to err and can quickly match the data inputted by the user with the relevant response and solutions, minimizing the chances of errors [73,74]. The study participants indicated the existence of barriers associated with seeking sexual reproductive health information that the AGILE chatbot can significantly address. Other studies corroborate the fact that there are few data available on sexual health, GBV, and related topics affecting service delivery in this topic [74,75,76]. Participants articulated a need for accurate and comprehensive information on these subjects, highlighting the chatbot’s potential to bridge information gaps. The users were also willing to engage the AGILE chatbot if its accuracy was guaranteed. Additionally, they expressed a desire for the chatbot to be integrated with human interactions, such as a toll-free number as a way to enhance the quality of services, provide reassurance, and guide survivors through necessary procedures and reporting.

In summary, this study contributes to the growing body of literature on using HCD in chatbot development to support survivors of GBV. It provides nuanced insights into user expectations and preferences regarding chatbots, particularly in the context of GBV, sexual and reproductive health, mental health information and service provision. Additionally, the study’s findings align with and reinforce the principles of user-centered design, emphasizing the importance of meeting user needs and expectations in the development of digital healthcare interventions.

The study had limitations considering that incorporating user preferences in product development is a relatively new idea in the Kenyan context. Adapting from conventional help-seeking approaches to the AGILE platform may take some time. Less technologically oriented users may experience challenges using the chatbot and may not benefit from its features. A significant part of the Kenyan population experiences poverty and compromised information technology infrastructure and may not have access to digital devices, or steady internet may hamper them from the benefits of using the AGILE chatbot.

## 5. Conclusions

The interaction with vulnerable populations, including adolescents, young women, and sexual and gender minorities, demonstrates the significance of user preferences in enhancing the availability and accessibility of vital GBV services. These vulnerable populations must frequently use digital platforms for learning, social interactions, and leisure (social media) Therefore, incorporating human-centered GBV interventions for these populations will be crucial in addressing common barriers to seeking care, including service provider attitude, lack of personnel trained to offer client-centered services, stigma and discrimination, long queues, and proximity of care facility. A virtual assistant that operates confidentially, around the clock, and understands different languages with features including sentiment analysis, multilingual users, spelling corrections, and translation will enhance communication, functionality, and accessibility of services. Adopting a human-centered approach in designing an effective chatbot with as many human features as possible is critical to increasing utilization to address GBV gaps by moving prevention and response services closer to hard-to-reach and vulnerable populations.

## Data Availability

Data used in the analyses for this study are available upon request from the corresponding author.
